# Supplementary material for: Folate receptor targeted nanoparticles containing niraparib and doxorubicin as a potential candidate for the treatment of high grade serous ovarian cancer
Source: Sci Rep. 2023 Feb 24;13:3226. doi: 10.1038/s41598-023-28424-3 (PMC9958112; doi:10.1038/s41598-023-28424-3)
Supplement: Supplementary file 1 — Supplementary Information. [file 41598_2023_28424_MOESM1_ESM.docx]

# Supplementary Information


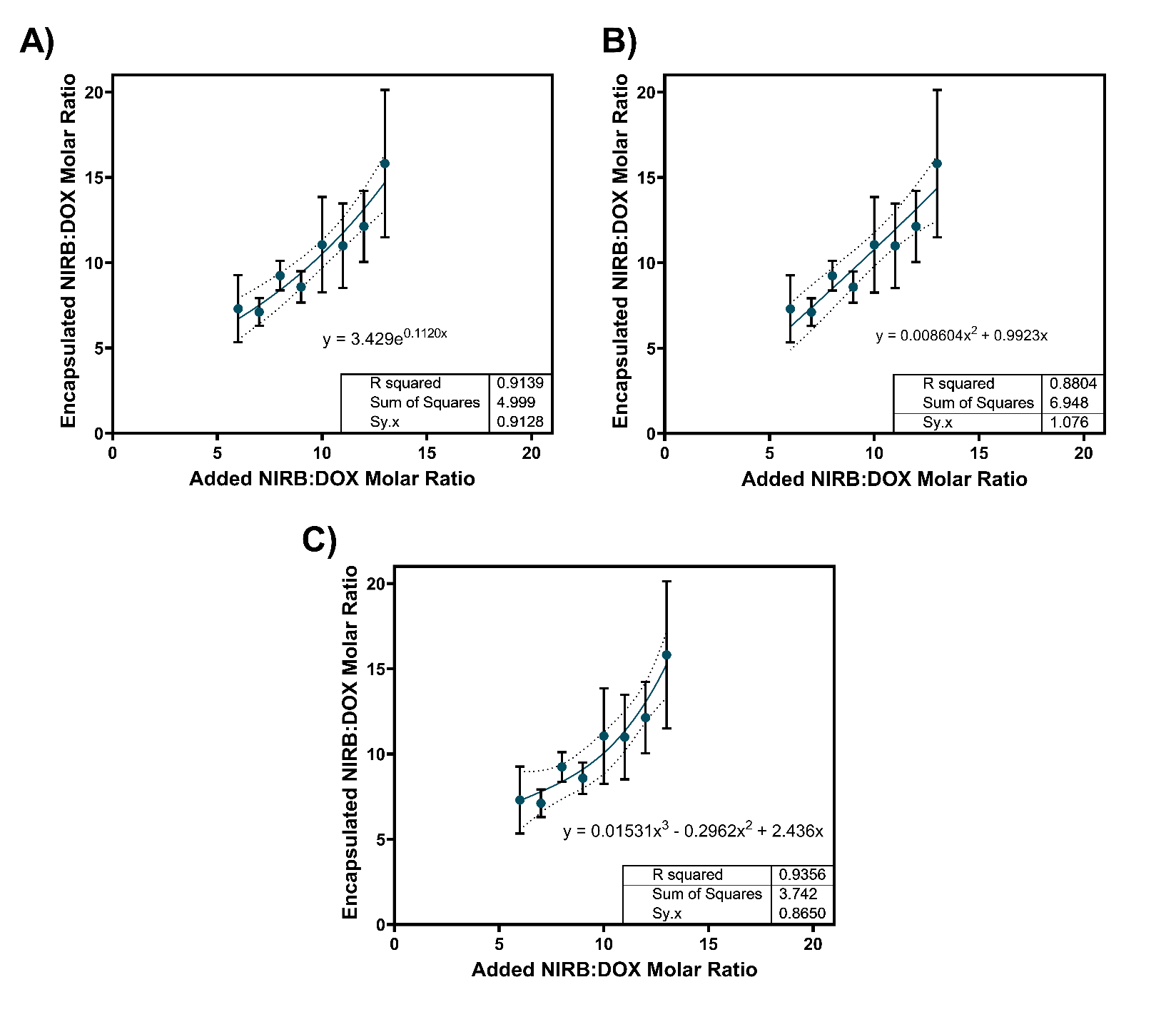


**Figure S1: Relationship between added NIRB and DOX relative to encapsulated NIRB and DOX expressed as A) exponential, B) second order polynomial, and C) third order polynomial models.** Individual experimental replicates are graphed as mean±SD with Sy.x (standard deviation of the residuals) selected as the appropriate goodness-to-fit indicator for non-linear regressions. The blue dotted lines indicate the 95% confidence intervals for each non-linear model. Note that the 95% confidence windows of predictions slightly widen at higher levels of encapsulated NIRB relative to DOX, suggesting poorer model reliability when loading liposomes with relatively higher NIRB content compared to DOX.


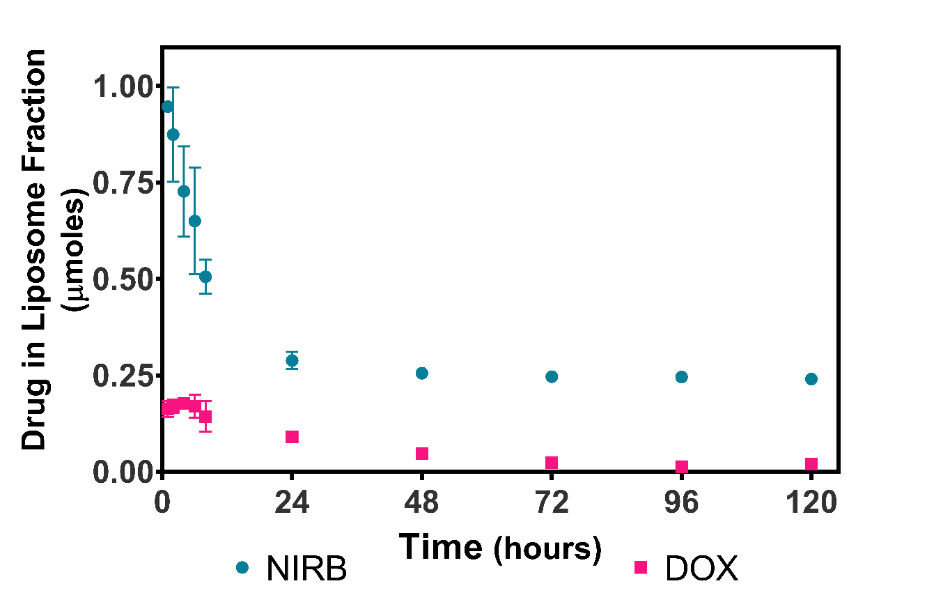


**Figure S2: *In vitro* release of NIRB and DOX from 300mM sodium citrate** **liposomes (NIRB-DOX-CIT) in BSA.** The y-axis shows the mean absolute value (μmoles) of NIRB and DOX remaining in the liposomes (±SD) within collected 100μL fractions when NIRB-DOX-CIT liposomes are subjected to biologically relevant (i.e., 50mg/mL of BSA pH 7.4, 37°C) sink conditions over five days. Unlike sodium citrate core liposomes encapsulating only NIRB (Supplementary Figure S3), dual-encapsulated NIRB-DOX-CIT liposomes exhibited rapid release of drug cargo within the first 24 hours (84.5±0.81% of NIRB and 61.9±6.49% of DOX at the 24-hour timepoint). It is hypothesized that while sodium citrate can adequately form stable ion complexes with either NIRB or DOX individually, the citrate anions are unsuitable for the entrapment of both NIRB and DOX simultaneously. Further investigation is required to elucidate the cause of this event. All experiments were completed as n=3.


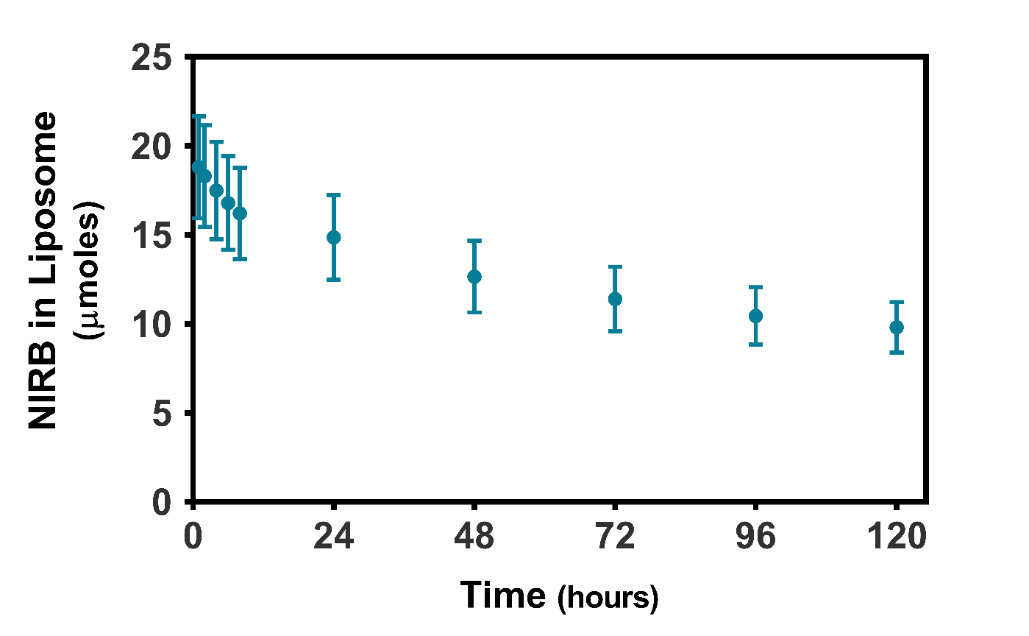


**Figure S3: *In vitro* release of NIRB from 300mM sodium citrate liposomes in BSA.** The y-axis shows the mean absolute value (μmoles) of NIRB remaining in all liposomes (±SD) when NIRB loaded sodium citrate core liposomes are subjected to biologically relevant (i.e., 50mg/mL of BSA, pH 7.4, 37°C) sink conditions over five days. Sodium citrate core liposomes encapsulating only NIRB exhibited sustained release over the five-day study (49.4±1.17% of drug was released at the 120-hour timepoint). All experiments were repeated as n=3.


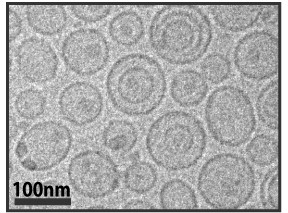


**Figure S4: Cryo-TEM images of folate conjugated, NIRB and DOX dual-encapsulated liposomes using sodium citrate as an entrapment agent (NIRB-DOX-CIT).** Note the lower levels of visually discernable intraliposomal dark areas, indicating a lack of electron dense deposits at the core. This is in direct contrast to NIRB-DOX-FA liposomes which use TEA_8_SOS as an entrapment agent, suggesting sodium citrate’s inability to facilitate the formation of solid drug co-precipitates. These observed distinctions are plausible rationale for the stark differences in *in vitro* release profiles between the presented formulations.


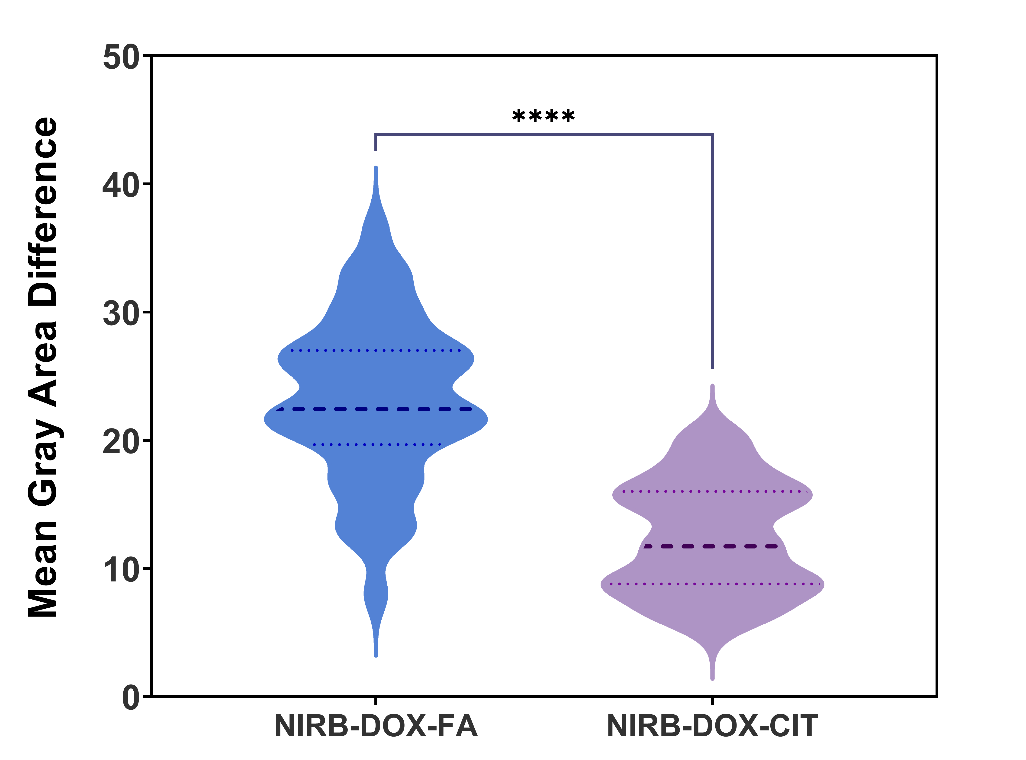


**Figure S5: Differences in mean gray area of the exterior versus interior of folate-targeted, NIRB and DOX dual-encapsulated liposomes using sodium citrate (NIRB-DOX-CIT) versus TEA_8_SOS** **(NIRB-DOX-FA) as an entrapment agent.** Mean gray area is defined as the average photo intensity divided by the number of pixels in a selected image area. Mean gray area was determined to quantify the darkened electron dense areas at the core of NIRB-DOX-CIT versus NIRB-DOX-FA liposomes. As such, statistically significant differences in mean gray area across the bilayer of NIRB-DOX-FA liposomes relative to NIRB-DOX-CIT liposomes, highlights the formation of electron dense drug deposits using TEA_8_SOS and not sodium citrate as an entrapment agent. Mean gray area of the liposomes was determined by ImageJ software for cryo-TEM images. Data is presented as differences in mean gray area (n=43, **** p<0.0001).

**Table S1: Heat map summary of A-B) DOX and C-D) NIRB dosages required to achieve 50% and 75% cell death within the corresponding combination compared to as monotherapy.** As shown, synergistic combinations trend towards higher-than-expected individual drug potency compared to either DOX or NIRB alone.

|  | **DOX Dose (nM); Fa= 0.5** | | | | | | | | | | | | | | | | | | | | |  |
| --- | --- | --- | --- | --- | --- | --- | --- | --- | --- | --- | --- | --- | --- | --- | --- | --- | --- | --- | --- | --- | --- | --- |
|  |  |  |  |  |  |  |  |  |  |  |  |  |  |  |  |  |  |  |  |  |  |  |
| **A)** | **NIRB:DOX** | **20:1** | | **10:1** | | **5:1** | | **2:1** | | **1:1** | | **1:2** | | **1:5** | | **1:10** | | **1:20** | | **DOX** | |  |
|  |  | **Mean** | **SEM** | **Mean** | **SEM** | **Mean** | **SEM** | **Mean** | **SEM** | **Mean** | **SEM** | **Mean** | **SEM** | **Mean** | **SEM** | **Mean** | **SEM** | **Mean** | **SEM** | **Mean** | **SEM** |  |
|  | **OVCAR8** | 45 | 7 | 47 | 7 | 71 | 6 | 68 | 5 | 65 | 8 | 86 | 23 | 78 | 5 | 78 | 10 | 70 | 11 | 75 | 7 |  |
|  | **HEYA8** | 42 | 6 | 37 | 4 | 43 | 4 | 51 | 5 | 43 | 5 | 74 | 23 | 60 | 8 | 57 | 3 | 47 | 6 | 54 | 2 |  |
|  | **PEO1** | 117 | 6 | 111 | 16 | 123 | 10 | 133 | 10 | 120 | 10 | 132 | 21 | 103 | 14 | 115 | 19 | 105 | 19 | 136 | 11 |  |
|  | **PEO4** | 223 | 39 | 190 | 47 | 220 | 20 | 210 | 17 | 226 | 30 | 327 | 47 | 247 | 19 | 274 | 34 | 237 | 21 | 241 | 26 |  |
|  | **DOX Dose (nM); Fa= 0.75** | | | | | | | | | | | | | | | | | | | | |  |
|  |  |  |  |  |  |  |  |  |  |  |  |  |  |  |  |  |  |  |  |  |  |  |
| **B)** | **NIRB:DOX** | **20:1** | | **10:1** | | **5:1** | | **2:1** | | **1:1** | | **1:2** | | **1:5** | | **1:10** | | **1:20** | | **DOX** | |  |
|  |  | **Mean** | **SEM** | **Mean** | **SEM** | **Mean** | **SEM** | **Mean** | **SEM** | **Mean** | **SEM** | **Mean** | **SEM** | **Mean** | **SEM** | **Mean** | **SEM** | **Mean** | **SEM** | **Mean** | **SEM** |  |
|  | **OVCAR8** | 173 | 19 | 190 | 22 | 231 | 20 | 243 | 16 | 223 | 18 | 288 | 60 | 246 | 19 | 241 | 21 | 243 | 33 | 252 | 24 |  |
|  | **HEYA8** | 117 | 13 | 126 | 11 | 136 | 18 | 142 | 11 | 132 | 9 | 187 | 32 | 157 | 13 | 152 | 10 | 141 | 13 | 153 | 10 |  |
|  | **PEO1** | 299 | 15 | 337 | 23 | 332 | 20 | 332 | 19 | 341 | 25 | 360 | 39 | 328 | 27 | 346 | 40 | 320 | 32 | 359 | 11 |  |
|  | **PEO4** | 733 | 58 | 712 | 66 | 749 | 51 | 775 | 29 | 823 | 11 | 1001 | 116 | 853 | 49 | 841 | 50 | 726 | 47 | 749 | 42 |  |
|  | **NIRB Dose (nM); Fa= 0.5** | | | | | | | | | | | | | | | | | | | | |  |
|  |  |  |  |  |  |  |  |  |  |  |  |  |  |  |  |  |  |  |  |  |  |  |
| **C)** | **NIRB:DOX** | **20:1** | | **10:1** | | **5:1** | | **2:1** | | **1:1** | | **1:2** | | **1:5** | | **1:10** | | **1:20** | | **NIRB** | |  |
|  |  | **Mean** | **SEM** | **Mean** | **SEM** | **Mean** | **SEM** | **Mean** | **SEM** | **Mean** | **SEM** | **Mean** | **SEM** | **Mean** | **SEM** | **Mean** | **SEM** | **Mean** | **SEM** | **Mean** | **SEM** |  |
|  | **OVCAR8** | 907 | 139 | 474 | 69 | 354 | 28 | 136 | 10 | 65 | 8 | 43 | 11 | 16 | 1 | 8 | 1 | 3 | 1 | 2902 | 198 |  |
|  | **HEYA8** | 833 | 111 | 374 | 41 | 217 | 19 | 102 | 9 | 43 | 5 | 37 | 12 | 12 | 2 | 6 | 0 | 2 | 0 | 6140 | 1054 |  |
|  | **PEO1** | 2333 | 116 | 1110 | 157 | 616 | 50 | 266 | 21 | 120 | 10 | 66 | 10 | 21 | 3 | 11 | 2 | 5 | 1 | 29306 | 2400 |  |
|  | **PEO4** | 4458 | 779 | 1902 | 465 | 1099 | 98 | 420 | 34 | 226 | 30 | 163 | 23 | 49 | 4 | 27 | 3 | 12 | 1 | 23298 | 3992 |  |
|  | **NIRB Dose (nM); Fa= 0.75** | | | | | | | | | | | | | | | | | | | | |  |
|  |  |  |  |  |  |  |  |  |  |  |  |  |  |  |  |  |  |  |  |  |  |  |
| **D)** | **NIRB:DOX** | **20:1** | | **10:1** | | **5:1** | | **2:1** | | **1:1** | | **1:2** | | **1:5** | | **1:10** | | **1:20** | | **NIRB** | |  |
|  |  | **Mean** | **SEM** | **Mean** | **SEM** | **Mean** | **SEM** | **Mean** | **SEM** | **Mean** | **SEM** | **Mean** | **SEM** | **Mean** | **SEM** | **Mean** | **SEM** | **Mean** | **SEM** | **Mean** | **SEM** |  |
|  | **OVCAR8** | 3513 | 402 | 1940 | 237 | 1209 | 137 | 546 | 65 | 277 | 68 | 246 | 130 | 100 | 52 | 71 | 47 | 80 | 69 | 13069 | 1009 |  |
|  | **HEYA8** | 2377 | 281 | 1292 | 127 | 723 | 130 | 318 | 39 | 163 | 37 | 156 | 78 | 68 | 38 | 51 | 36 | 44 | 38 | 20781 | 1753 |  |
|  | **PEO1** | 6053 | 312 | 3443 | 277 | 1728 | 134 | 745 | 83 | 421 | 96 | 282 | 121 | 147 | 86 | 112 | 78 | 97 | 82 | 121738 | 16490 |  |
|  | **PEO4** | 14846 | 1200 | 7267 | 682 | 3864 | 194 | 1740 | 188 | 984 | 158 | 784 | 336 | 366 | 202 | 227 | 140 | 171 | 134 | 52102 | 3388 |  |
